# Preservation of neurocognitive function in the treatment of brain metastases

**DOI:** 10.1093/noajnl/vdab122

**Published:** 2021-11-27

**Authors:** Michael W Parsons, Katherine B Peters, Scott R Floyd, Paul Brown, Jeffrey S Wefel

**Affiliations:** 1 Pappas Center for Neuro-Oncology, Department of Psychiatry, Massachusetts General Hospital, Boston, Massachusetts, USA; 2 Preston Robert Tisch Brain Tumor Center, Department of Neurosurgery, Duke University Medical Center, Durham, North Carolina, USA; 3 Department of Radiation Oncology, Duke University School of Medicine, Durham, North Carolina, USA; 4 Department of Radiation Oncology, Mayo Clinic, Rochester, Minnesota, USA; 5 Department of Neuro-Oncology, The University of Texas MD Anderson Cancer Center, Houston, Texas, USA; 6 Department of Radiation Oncology, The University of Texas MD Anderson Cancer Center, Houston, Texas, USA

**Keywords:** brain neoplasms, cancer, cognition, neuropsychology, survivors

## Abstract

Neurocognitive function (NCF) deficits are common in patients with brain metastases, occurring in up to 90% of cases. NCF deficits may be caused by tumor-related factors and/or treatment for the metastasis, including surgery, radiation therapy, chemotherapy, and immunotherapy. In recent years, strategies to prevent negative impact of treatments and ameliorate cognitive deficits for patients with brain tumors have gained momentum. In this review, we report on research that has established the efficacy of preventative and rehabilitative therapies for NCF deficits in patients with brain metastases. Surgical strategies include the use of laser interstitial thermal therapy and intraoperative mapping. Radiotherapy approaches include focal treatments such as stereotactic radiosurgery and tailored approaches such as hippocampal avoidant whole-brain radiotherapy (WBRT). Pharmacologic options include use of the neuroprotectant memantine to reduce cognitive decline induced by WBRT and incorporation of medications traditionally used for attention and memory problems. Integration of neuropsychology into the care of patients with brain metastases helps characterize cognitive patterns, educate patients and families regarding their management, and guide rehabilitative therapies. These and other strategies will become even more important for long-term survivors of brain metastases as treatment options improve.

As treatments for systemic cancer have improved, the proportion of patients experiencing metastatic disease to the central nervous system continues to increase. The development of CNS metastases, estimated to occur in up to 30% of patients with systemic cancer,^1^ is a devastating event portending a dire prognosis for overall survival and threatening the independence and identity of the patient. The most common metastases to the brain are from lung cancer, breast cancer, melanoma, and renal cell cancer.^2^ These patients face difficult treatments, including neurosurgery, radiation therapy, and additional rounds of systemic therapy.

Loss of neurocognitive function (NCF), which has been shown to occur in as many as 90% of patients with brain metastases,^3^ can be caused the tumor itself and by the treatments applied to both systemic and CNS disease. Numerous aspects of NCF, including memory, processing speed, and executive function, are disrupted in patients with brain metastases prior to any CNS directed treatment,^4–6^ and successful treatment of brain metastases can lead to improvement in neurologic symptoms attributable to the disease itself and forestall the inevitable decline associated with tumor progression. Treatment of brain metastases requires a multimodal approach and may include radiation therapy, surgery, chemotherapy, and newer techniques such as immunotherapy or laser interstitial thermal ablative therapy. While the goal of these therapies is to improve progression-free and overall survival, they can also cause brain injury leading to neurocognitive dysfunction. Understanding the mechanisms of how these therapies can damage healthy brain tissue is vital for educating our patients and their caregivers about these impairments and developing strategies to mitigate or prevent the injury.

Declines in NCF are associated with a significant impact on the individual’s quality of life (QOL), including losing the ability to perform a job, safely operate a motor vehicle (estimated at over 40% in one study^7^), take care of a family, manage a household, and even care for oneself.^8–10^ In a study examining the relationship between NCF, QOL, and activities of daily living (ADLs),^11^ NCF deficits were strongly associated with problems with ADLs and QOL in patients with brain metastases who were treated with whole-brain radiation therapy (WBRT). After treatment, declines in NCF preceded and predicted ADL and QOL deterioration, which occurred in a substantial percentage of brain metastasis patients (40% and 34%, respectively). The NCF deficits experienced by patients with brain metastases constitute a threat to the individual in a way that is distinct from the other symptoms of cancer, striking at their identity and sense of independence,^12^ undermining their sense of productivity and meaning in life, financial security, and potentially limiting their access to care if health insurance coverage through an employer is lost. Many patients with brain metastases have a poor prognosis, whereas others are benefiting from new treatments that are extending progression-free and overall survival. In either case, it is incumbent upon treatment providers to optimize the patient’s QOL, providing the best possible treatment of disease and minimizing the deleterious impact of those treatments on NCF.

## Radiation Therapy

### Mechanisms of Brain Injury and Associated Neurocognitive Function Decline

When considering radiation therapy for the treatment of brain metastases, one can focus on delivery techniques. Classically, WBRT has been employed for disseminated metastases or prophylactically for cancers such as small cell lung carcinoma. As it has been recognized that WBRT can lead to moderate to severe neurocognitive dysfunction, more modern treatment paradigms have shifted to using stereotactic radiosurgery (SRS) to target solitary and oligo-metastatic disease, which reduces the risk of NCF impairment without compromising progression and survival endpoints.^4^

RT fundamentally leads to DNA damage via the generation of reactive oxygen species. In addition to the production of DNA double-strand breaks and single-strand breaks in tumor cells, all components of healthy cells are damaged by radiation. The primary mechanisms of this damage are likely activation of inflammation of the neural tissue along with activation of microglia, which represent CNS-derived macrophage-like cells.^13^ Key mediators of neurotoxic inflammation include tumor necrosis factor-alpha (TNF-α) and interleukin-8 (IL-8). This inflammation can occur during or immediately after RT, leading to the acute cognitive side effects and the chronic white matter loss seen in patients exposed to RT with late loss of microglia.^14^ Radiologically, these phenomena can manifest as acute inflammation during radiation therapy and widespread leukoencephalopathy years after radiation therapy is completed.^15^ In concert with increased neuroinflammation, radiation disrupts the normal functioning of neural progenitor cells, particularly in the region of the hippocampus, a neural structure of the temporal lobe that is critical for learning and memory.^16^

Historically, neurocognitive decline has been described as occurring at different stages during and after treatment with WBRT: acute changes that occur within days of initiation of treatment, subacute changes evolving in the weeks after treatment through the first few months after completion, and chronic/progressive changes, which generally begin to appear about 6 months after treatment and lead to inexorable deterioration. More recently, randomized trial data from patients undergoing treatment for brain metastases with or without the use of WBRT have demonstrated that many patients show declines in memory and executive functioning by 4 months after treatment.^4,17^ Clinically, these deficits may manifest as “forgetfulness,” with patients requiring lists and reminders, and often dependence on caregivers to compensate for these deficits and their impact on ADLs. As a considerable minority of patients are long-term survivors following WBRT, strong data are lacking regarding more long-term neurocognitive outcomes, however, clinically the usual course is of plateauing or slow decline in memory, with more dependence on caregivers for ADLs.

### Prevention and Treatment of Neurocognitive Decline From RT

**Figure 1.**
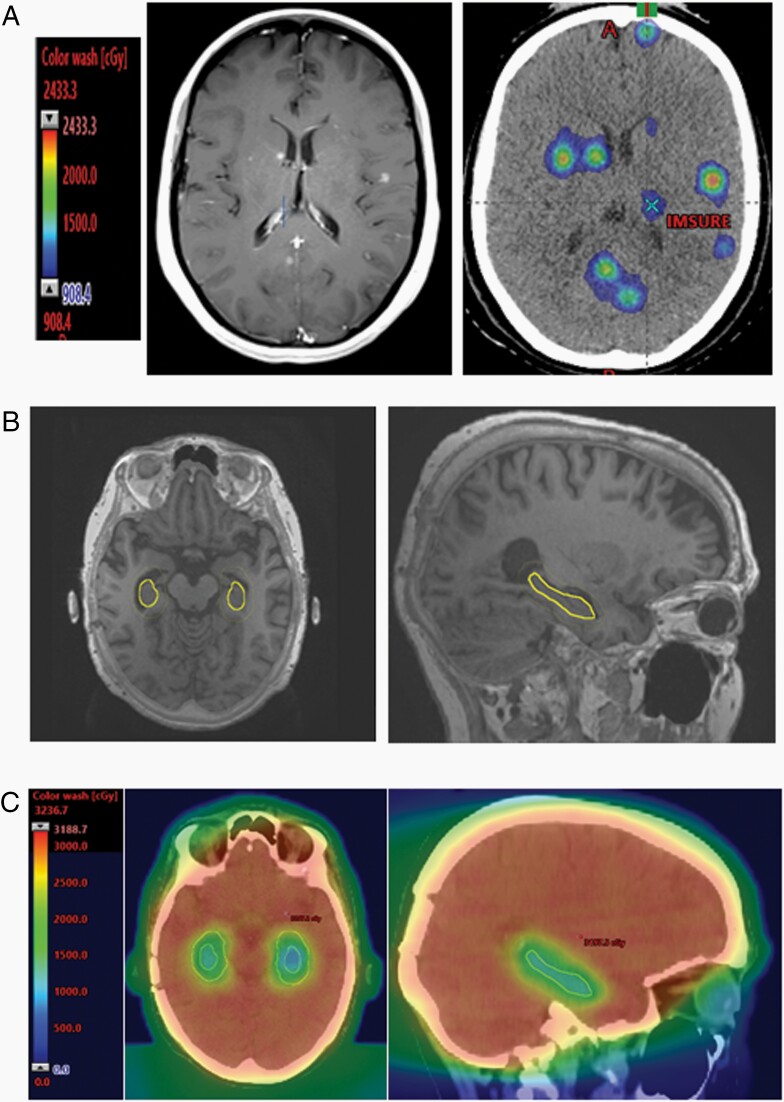
(A) Stereotactic radiosurgery (SRS) radiation treatment plan for multiple metastases shown on T1 contrasted MRI on left with single fraction doses in color wash dose distributions on right. (B) Hippocampal avoidant whole-brain radiotherapy (HA-WBRT): The hippocampal avoidance region (yellow) is generated by expanding the hippocampal contour (thin yellow) by 5 mm. (C) HA-WBRT (30 Gy in 10 fractions) color wash dose distributions are shown on representative axial and sagittal images.

Recognizing the importance of RT in the management of brain metastases, diverse strategies have been explored to mitigate the cognitive morbidity of treatment. The goal of these strategies has been to achieve equivalent disease control while reducing cognitive side effects by using strategies such as focal irradiation, avoidance of critical neural structures, and neuroprotectant therapies. Promising results from early studies suggested that individuals with brain metastases treated with focal RT such as SRS (Figure 1A) have better neurocognitive outcomes compared to those treated with SRS plus WBRT.^17^ The pivotal multicenter phase III clinical trial^4^ included 213 individuals with 1–3 brain metastases. Subjects were randomized to either receive SRS or WBRT plus SRS. Patients who received only SRS had better NCF outcomes both at an early time point (the proportion of patients experiencing cognitive deterioration at 3 months was 19% after SRS alone compared to 46% with SRS plus WBRT) and for long-term survivors (at 12 months, 43% of WBRT plus SRS patients had deterioration on an executive function task, compared with 0% of the SRS alone patients). Although the WBRT group had lower rates of brain metastasis recurrence (distant control), there was no significant difference in overall survival (hazard ratio, 1.02; 95% CI, 0.75–1.38; *P* = .92).

Given the frequency of memory impairment after WBRT^18^ as well as the role of the hippocampus in neuronal regeneration and plasticity, an additional neuroprotective strategy has been to modify treatment to selectively avoid the hippocampal region (hippocampal avoidance; HA-WBRT; see Figure 1B and C).^19,20^ A phase II single-arm study evaluating HA-WBRT found improved outcomes on tests of memory performance in comparison to an expected level of impairment based on historical outcomes.^21^

The use of pharmacological agents to protect against radiation-induced cerebral injury has been an additional strategy to reduce neurocognitive morbidity. Memantine, an *N*-methyl-d-aspartate (NMDA) receptor antagonist that reduces harmful excessive stimulation of NMDA receptors has been shown to reduce neurotoxicity of radiation therapy.^22,23^ Brown et al.^24^ found those who received memantine during WBRT showed longer time to cognitive decline than those who did not receive memantine. The memantine group also had stronger performance on measures of executive functioning 16 weeks later and better processing speed and delayed recognition at 24 weeks.^24^ More recently, the combined use of memantine and HA-WBRT^25^ was demonstrated to further reduce the frequency of NCF decline in a large phase III clinical trial.

## Surgery

### Mechanisms of Brain Injury and Associated Neurocognitive Function Decline

The impact of a metastatic tumor on the CNS relates to the overall burden of disease in the brain, most accurately quantified as the overall volume of brain disease.^5^ Concomitant neurologic sequelae, including edema, seizures, and headaches, can also contribute to NCF dysfunction. Furthermore, the medications to treat these complications can have adverse effects on neuronal function. While neurological injury leading to NCF decline is possible during neurosurgical procedures^26^ due to trauma to the local healthy brain tissue, to the extent that resection reduces mass effect, edema, or disruption of CSF flow, surgery can lead to improved performance status maintained over a longer period of time.^27^ Similar to radiation-induced damage, studies have found that after brain surgery, rats and mice demonstrate increased neural tissue inflammation with resulting induction of TNF-α and IL-8.^28,29^ Additionally, brain-derived neurotrophic factor (BDNF) is reduced, with a consequent decrease in hippocampal neurogenesis. These changes are accompanied by NCF impairments in murine behavioral tests.^30^ Xin et al. have shown that by inhibiting proinflammatory signaling pathways, in particular nitrous oxide (NO) pathways, one can rescue postoperative cognitive dysfunction in mice and rats.^31^ Developing methods to limit neuroinflammation after surgery has the opportunity to provide protection against and mitigation of neurocognitive dysfunction.

### Minimizing the NCF Risk of Neurosurgery in Patients With Brain Metastases

Neurocognitive risk of surgery is greatest when brain metastases arise near eloquent areas, particularly speech/language and memory-related areas in the dominant hemisphere.^32^ Technological advances, such as intraoperative mapping of cognitive function during awake craniotomy, provide an opportunity for the monitoring of NCF during the procedure. Although awake craniotomy is more commonly employed in the resection of primary brain tumors, a recent review found that awake craniotomy for brain metastases was a viable option to reduce cognitive morbidity.^33^ The review showed that surgery in/near eloquent cortex leads to increased risk of postoperative neurocognitive deficits as compared with surgeries farther from eloquent regions; however, 73% of patients undergoing awake craniotomy were not found to have a decline on a brief bedside neurologic exam conducted by the surgeon. Of those who had a decline in the acute postoperative period, 96% showed subsequent improvement and recovery.

Laser interstitial thermal therapy (LITT) involves neurosurgical stereotactic placement of a laser probe that kills tumor tissue with heat.^34^ Heating above 50°C leads to cell death. LITT is now becoming more widely used in patients with brain metastases. Similar to the injury induced by traditional neurosurgical procedures, anatomical location and proximity to eloquent areas or areas involved in cognition are vital to understand how LITT could impact NCF. One can glean information on long-term cognitive data by looking at the use of LITT in epilepsy patients. Small nonrandomized studies point out that memory decline can occur in patients with dominant medial temporal lobe epilepsy who underwent LITT involving the hippocampus,^35^ but these patients were spared the language declines often seen in patients who undergo standard resective surgery for this condition. In patients with brain metastases, where the target of LITT is not functional neural tissue, the hope is that this approach could lead to reduced neurocognitive morbidity in difficult to reach areas of the brain compared with resective surgery. A study of 39 patients, 20 of whom had brain metastases (19 more had radiation necrosis) by Ahluwalia et al. demonstrated no reduction in neurocognitive performance.^36^ The role of LITT in patients with brain metastases continues to be explored. Balancing the risks and benefits of surgery along with application of these new techniques in eloquent areas will continue to be the aim for neurosurgical procedures in brain metastases patients.

## Systemic Therapies

### Chemotherapy

In concert with other therapeutics, use of systemic agents in patients with brain metastases is expanding rapidly with the list of FDA approved targeted therapies enlarging. The trend for chemotherapy in treatment of brain metastases lies prominently with targeted agents. Regardless, there is continued use of agents such as methotrexate, capecitabine, and paclitaxel, all of which have been found to affect NCF. For example, neurotoxicity of methotrexate therapy has been well demonstrated in adult CNS lymphoma patients^37^ and adverse neurocognitive outcomes have been found in survivors of treatment for childhood leukemia.^38^ Capecitabine effects on neurocognition have been reported but are generally mild,^39^ while paclitaxel effects have also been of concern, given anti-microtubule mechanism and known association with peripheral neuropathy and acute encephalopathy.^40^ An extensive literature demonstrates the neurotoxicity of these and many other traditional chemotherapy agents (for a recent review, see Dietrich^41^), which may manifest as acute or subacute neurocognitive syndromes or more subtle cognitive deficits that are longer lasting (eg, *chemobrain*). Multiple mechanisms have been proposed for these effects, including inflammatory mechanisms, direct cellular toxicity, myelin damage, and loss of hippocampal neurogenesis.^42^ Patients with brain metastases receiving high-dose or intrathecal methotrexate are further at risk for the development of methotrexate-induced leukoencephalopathy, demonstrated by NCF impairment in the setting of T2/FLAIR hyperintensities on magnetic resonance imaging, particularly when combined with radiation therapy.^43^ In preclinical rat and murine models, exposure to methotrexate (with or without 5-fluoruracil) leads to NCF dysfunction, which was attributable to loss of neurons in the hippocampus and frontal lobes.^44^ More recent work demonstrates that damage to pathways involved in oligodendrocyte integrity and adaptive myelination via BDNF signaling is responsible for cognitive impairment in mice exposed to methotrexate.^45^

### Hormonal Therapy

While hormone-based therapies are not classic chemotherapy, these treatments are commonly used in the treatment of breast and prostate cancer and may contribute to cognitive impairment.^46^ Theoretically, these effects reflect the ubiquitous expression of estrogen and androgen receptors in key areas of the brain involved in cognition, such as prefrontal cortices and the hippocampus.^47^ A recent review of this literature^46^ concluded that there is evidence of cognitive impairment in patients with breast or prostate cancer after treatment with hormonal therapies. A longitudinal prospective study followed women with breast cancer who either were or were not treated with hormonal therapies found no difference in cognitive symptoms or performance up to 6 years posttreatment,^48^ though this analysis was limited to group comparisons and may have missed possible individual differences in response to therapy. In men undergoing androgen deprivation therapy (ADT) for prostate cancer, a longitudinal prospective study showed no differences in cognitive performance over a 3-year period between patients who were treated with ADT, patients who did not need such treatment, and healthy controls.^49^ However, a similar study found increased risk for cognitive decline in patients treated with ADT and identified a genetic risk factor that appeared to markedly increase risk in a subset of patients.^50^ Thus, it appears that there are cognitive risks associated with hormone therapies and additional research is needed to identify the relevant risk factors and longer term outcomes.

### Immunotherapy

Immunotherapy has revolutionized the treatment of patients with melanoma and other cancers that regularly send metastases to the central nervous system; however, encephalitis due to autoimmune induced inflammation in the brain can lead to both acute and chronic neurologic impairments.^51^ The long-term implication for NCF function is best determined when one considers concomitant use of radiation therapy. McGinnis et al. developed preclinical models in mice exposed to immunotherapy, particularly immune-checkpoint inhibitors (ICIs) with and without concomitant radiation therapy.^52^ All mice that received the combination of immunotherapy and radiation developed cognitive impairment and notably, this cognitive impairment was in the setting of tumor control. Mechanistically, microglia appeared to be activated by immunotherapy, with or without concomitant radiation.

## Evaluation and Management of Cognitive Problems in Patients With Brain Metastases

### Identification of Risk for Cognitive Decline

Despite efforts to reduce the cognitive risks of therapy detailed above, many patients with brain metastases will experience cognitive symptoms during the course of their disease, requiring a comprehensive clinical strategy for management.^3^ Identification of individuals at greatest risk of NCF morbidity is an important aspect of treatment decision making. Advanced age and a higher degree of pretreatment leukoencephalopathy are both associated with a greater risk of cognitive dysfunction in patients with brain metastases who received WBRT,^53,54^ suggesting that health of the underlying brain tissue contributes to NCF risk in these patients. Researchers have initiated studies into the genetic factors associated with the risk of NCF dysfunction in patients with primary brain tumors and those who received treatment for CNS and non-CNS cancers. Variations on a theme that implicates apolipoprotein E (APOE), a gene related to the risk of Alzheimer’s disease, are present in the literature pertaining to cancer related cognitive impairment (CRCI) in breast cancer patients.^55^ Unfortunately, the literature is at odds as to whether APOE genotype is meaningful in predicting risk of CRCI. Preliminary work suggests that those with a high-risk APOE genotype experience greater cognitive decline when they undergo WBRT than do those with a lower risk genotype.^56^ Correa et al.^57,58^ showed that specific SNPs in catechol-*O*-methyl transferase (COMT), BDNF, and dystrobrevin-binding protein 1 (DTNBP1) genes can be associated with dysfunction in a myriad of cognitive domains. How these translate specifically to patients with brain metastases remains to be determined.

### Evaluation and Monitoring

Monitoring NCF in the neuro-oncology clinic is difficult for clinicians because brief screening measures, such as the Mini-Mental State Examination (MMSE^59^) and the Montreal Cognitive Assessment (MoCA^60^) have only modest ability to detect symptoms in brain metastasis patients.^61,62^ Thus, a careful clinical inquiry regarding these symptoms is an important first step in assessing cognitive function during clinic visits. Self-report surveys can be used to inquire about subjective NCF changes in the context of QOL assessment with scales such as the Functional Assessment for Cancer Therapy-Brain (FACT-Br^63^) and the European Organization for Research and Treatment of Cancer Quality of Life Scale (EORTC-QLQ-C30^64^), including newly developed metrics that are moderately correlated with cognitive complaints.^65^ Neuropsychological (NP) evaluation is the most sensitive method of identifying cognitive dysfunction in patients with cancer, and specifically sensitive tests has been recommended by the International Cognition and Cancer Task Force (ICCTF).^66^ Neuropsychological evaluations have been flexibly integrated in the neuro-oncology clinic, including in metastatic brain tumor boards.^67^ These evaluations, often abbreviated to minimize burden on the patient,^68^ are sensitive to NCF changes and can detect progression of brain metastases prior to MRI.^69^ Integrating NP evaluations in the care of patients with brain metastases provides an understanding of NCF and related symptoms, recommendations for treatment, and guidance to the patient and family and is recommended in the most recent guidelines issued for CNS cancers by the National Comprehensive Cancer Network (NCCN; Section Brain E).^70^

### Pharmacotherapy

Medications used to treat cognitive symptoms have been trialed in brain tumor patients. In addition to the neuroprotectant role of memantine detailed above, pharmacologic agents used in the treatment of memory impairment and attention deficits in other neurologic populations have been studied. Most of this research has been in patients with primary brain tumors, but a few studies have also included patients with brain metastases.

An early study of the memory enhancer donepezil in a mixed group of patients with brain tumors had only one brain metastasis patient at baseline, who failed to complete the follow-up assessments,^71^ illustrating the difficulties of studying treatment outcomes in this patient population with such dismal survival. The largest study of donepezil in patients with brain tumors (*n* = 198) was a randomized placebo-controlled trial that included 53 patients (25%) with brain metastases^72^ and measured cognitive effects at 12 and 24 weeks of treatment. In the 74% of patients who completed follow-up visits (% of brain metastases not reported), there were subtle indications of a treatment effect on one measure of recognition memory.

Attention-enhancing medications, such as methylphenidate and modafinil, have been evaluated in mixed groups of brain tumor patients, though these studies too have largely excluded patients with brain metastases. Although early studies of this approach suggested some benefits,^73^ randomized placebo-controlled trials failed to replicate the findings, including the only study to include patient with brain metastases,^74^ suggesting that expectancy effects may play a significant role in the experience of patients prescribed these medications. It should be noted that this study evaluated fatigue, rather than cognitive function, as the primary endpoint.

### Rehabilitative Therapy

Cognitive rehabilitation is the use of therapeutic strategies to minimize the impact of NCF deficits on everyday functioning and/or improve cognitive function, which may include education in compensatory strategies as well as massed practice of cognitive exercises intended to provide neurocognitive stimulation. The majority of these studies have evaluated cognitive rehabilitation in patients with primary brain tumors and have suggested some benefit to those patients who receive training in specific cognitive strategies, such as the use of mnemonic strategies for memory problems^75^ and goal management training for executive function problems.^76^ Other approaches, such as combining the training of compensatory strategies with “cognitive exercise” activities have shown at least partial benefit in randomized controlled studies.^77^ Studies using remote methods (eg, telephone, computer) to deliver rehabilitation have also shown promise,^78^ including a method for cognitive stimulation leading to improved NCF test performance.^79^ To date, only two cognitive rehabilitation studies have included patients with brain metastases, both of which used variations of cognitive exercise training^79,80^ These small studies reported positive impacts of cognitive training but are limited to some extent by lack of a control group^80^ and small sample size.^79^ While these studies are opening doors for new methods, it has yet to be demonstrated that improvements on NCF tests or computerized exercises translate to benefits in the real world. Additional approaches to improve cognition in patients with brain tumors have included physical rehabilitation,^81^ which demonstrated a positive effect on MMSE scores in patients with brain tumors (including metastatic) in the weeks after surgery. There are hopes that other strategies such as exercise^82^ and herbal strategies^83^ may prove to be helpful, though the prevailing view in the field is that findings are too preliminary to form the basis of recommendations at this time.^84^

## Future Directions

The continued need for radiation therapy in the treatment of brain tumors and the increasing prevalence of brain metastases drives research into improving NCF for patients receiving brain radiotherapy. Model systems spanning the in vitro and in vivo spaces, including conventional and 3D culture systems, lend themselves to exploratory studies for neuroprotectors, while small animal radiation platforms and adaptations of clinical radiation therapy equipment^85,86^ enable in vivo validation and testing of candidate genes and drugs, as well as histopathology and imaging studies.^87–89^ Perhaps most importantly for preclinical research, elegant work to refine behavioral and neurocognitive testing in laboratory animals, often aided by complementary research from fields such as Alzheimer’s disease, developed assays such as the novel object recognition test and Morris water maze and correlative functional tests such as roto-rod to presage neurocognitive endpoints in humans.^90,91^

Numerous clinical trials are currently studying a wide range of approaches to improve cognitive outcomes in brain metastasis patients (see Table 1). On the drug discovery front for neurocognitive preservation in patients receiving RT, promising results from preclinical studies of several classes of small molecules have led to subsequent clinical trials, while more recent results hold promise for the future. Preclinical strategies to block the cytotoxic effects of radiation-induced NMDA channel activation contributed to the successful use of memantine.^24^ Combinations of memantine with AMPA receptor inhibitors are now planned for patients with primary brain tumors receiving RT^92^ and could soon extend to patients with brain metastases. Recently, development of manganese porphyrin compounds that alter the redox biology of mitochondria has generated interest as possible dual tumor radiosensitizers and normal tissue radioprotectors.^93^ Preclinical findings of preserved tumor control and neuroprotection with enhanced cognitive function following irradiation in mice spawned clinical trials in several cancer types, including brain metastases^87^ (NCT03608020). The role of GSK-3beta inhibition as a general neuroprotection strategy that prevents radiation necrosis^88^ is also exciting, and some clinical trial data exist for tideglusib in Alzheimer’s disease patients.^94,95^ Many other exciting data for novel drugs that target pathways such as hedgehog signaling^96^ and modulation of the complement cascade^97^ also show potential to improve neurocognitive outcomes in brain metastasis patients receiving radiotherapy.

**Table 1.** Current Clinical Trials for Neurocognitive Improvement in Brain Metastasis Patients

ClinicalTrials.gov #	Type	Summary
NCT04343157	Phase II single arm	Advanced MRI imaging to track radiation dose to critical structures and correlate to NCF
NCT03303365	Phase II single arm	Treatment of multiple metastases with Cyberknife device and imaging with MPRAGE or SPACE MRI: following cognitive outcomes
NCT0705548	Phase I	Dose escalation with fractionated SRS following cognitive outcomes
NCT03608020	Phase II randomized	Trial of manganese porphyrin BMX-001 to enhance NCF in brain metastasis patients receiving whole-brain radiotherapy
NCT04395339	Phase III	Trial of monosialotetrahexosy ganglioside (GM1) to preserve NCF in whole-brain radiotherapy patients
NCT03223922	Phase II single arm	Sparing of the genus of the corpus callosum in whole-brain radiotherapy patients
NCT03550391	Phase III	Comparison of WBRT to SRS for patients with 5–15 brain metastasis including neurocognitive endpoints

Abbreviations: NCF, neurocognitive function; SRS, stereotactic radiosurgery; WBRT, whole-brain radiotherapy.

Another strategy toward improving neurocognitive outcomes in patients receiving brain-directed RT leverages advanced radiation therapy techniques. Some of these techniques are in use currently and involve precise control over radiation dose deposition such that anatomical regions of the brain are spared damage. As noted above, HA-WBRT and SRS have shown neurocognitive benefit^4,25^ and further technical advances in the administration of SRS simultaneously to multiple target lesions promises to expand this technique for more patients with a high burden of brain metastases (NCT02886572). Upcoming trials will further differentiate the advantages of these approaches, and results are anxiously awaited (NCT03550391). While these techniques utilize the most advanced radiation therapy technologies that are in current clinical use, a newer technique has recently emerged that seeks to maximally exploit the fundamental differences in radiation biology responses that distinguish tumor from normal tissue. “Flash” radiation therapy employs ultra-high-dose rate radiation delivery (40–100 Gy/s) to harness a theoretical difference in normal tissue responses to radiation that has implications for radiotherapy to multiple areas of the body, including the brain.^98,99^ Although this approach is not yet available for widespread use, the first clinical tests of this technology and development of clinical instruments look promising.^100,101^

Lastly, biotechnology strategies that push the limits of current science focus on radiation-induced loss of neural stem cells in the hippocampus.^16^ Neural stem cell transplantation is theoretically possible, and with current stem cell technologies one can contemplate autotransplant of a patient’s own induced neural stem cells. Preclinical studies indicate that this approach could be beneficial^102,103^ and might be warranted in the increasingly plausible case that long-term cancer control in brain metastasis patients is attainable.

## Summary

Neurocognitive sequelae are an unfortunate reality for most patients with brain metastases, which can be caused by the metastatic tumors, treatment for the systemic disease, and treatment directed at the brain. Numerous advances over the past two decades, including neurosurgical techniques, focal delivery of radiotherapy, and neuroprotectant strategies, have reduced the negative impact on the brain. Integration of NP assessment in the routine care of patients with brain metastases allows for monitoring of cognitive outcomes and tailoring of treatment. Rehabilitative therapies and pharmacologic treatment of cognition are useful options for patients. As therapeutic options for cancer and brain metastases continue to improve, the focus on neurocognitive outcomes of the long-term survivors will become even more important.

## Case Example

**Figure 2.**
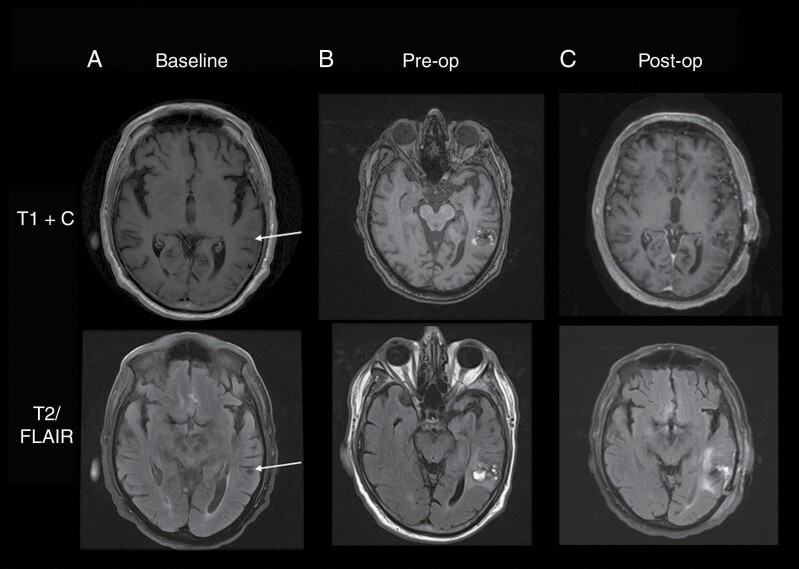
Axial T1-weighted contrast enhanced (T1 + C) and T2/FLAIR images for the patient described in the case example. (A) Images at the time of initial brain metastasis development (visible as only a very faint dot of increased signal on the T1 + C image, (B) at the time of progression approximately 1 year post-SRS treatment, and (C) postoperative imaging.

The following case example illustrates the multiple opportunities to integrate many of the techniques we have described in the clinical care of a patient with brain metastases to optimize cognitive outcome. The patient, a 67-year-old Caucasian man, initially developed a mass on the left upper back and underwent resection, with pathology confirming malignant melanoma. He had been treated with combination immunotherapy (ipilimumab + nivolumab) for approximately 3 months when he developed altered mental status. Brain imaging at the time was unrevealing, and the patient was suspected to be experiencing ICI-related encephalitis. He underwent treatment with intravenous immunoglobulin and neuro-rehabilitation during a 2-month hospitalization and ultimately recovered, though experienced a slightly reduced level of cognitive functioning compared with his normal baseline. ICI therapy was discontinued. Unfortunately, about 6 months later, surveillance brain imaging showed a subcentimeter enhancing lesion in the left lateral temporal lobe, which was felt to represent melanoma metastasis (Figure 2A). Based on the literature demonstrating adequate local control and reduced cognitive morbidity with SRS as opposed to WBRT, the patient was treated with single fraction SRS (18 Gy).

**Figure 3.**
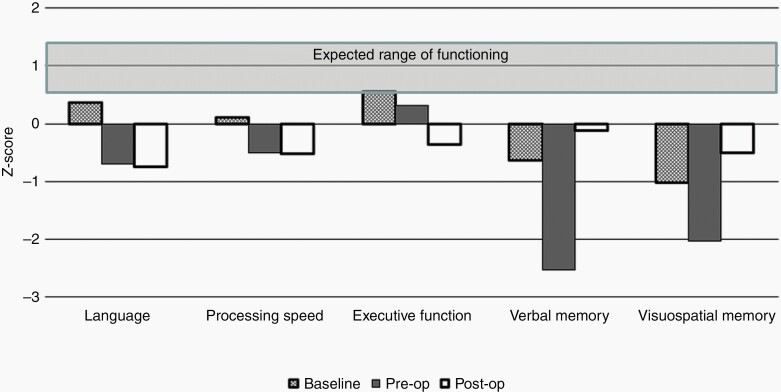
Graphical representation of neuropsychological test data in multiple domains, reported as standard scores compared with healthy controls matched for age and, where appropriate, education level. A *Z*-score of 0 represents the middle of the average range. Based on premorbid estimates of functioning, the patient’s expected level of functioning was above average. The initial evaluation showed slight reductions from expected levels of functioning. At the time of tumor progression, the patient showed a substantial decline in recent memory for both verbal and visuospatial information, with more subtle declines in other domains. Postoperative testing showed a marked improvement in memory performance, with return to the baseline level of functioning.

At the time of SRS, the patient reported persistent changes in his cognitive function compared with his usual baseline. Neuropsychological evaluation was requested at that time. During the interview, the patient reported difficulties with concentration and the ability to hold information in mind while multitasking (eg, working memory). He also described deficits in recent memory, such as forgetting conversations or things that he had agreed to do. He and his wife felt that these problems had been present since he recovered from ICI encephalitis and had not changed significantly since the new development of brain metastasis or SRS treatment of that lesion. The NP evaluation demonstrated that the patient was a man of above average premorbid ability who was experiencing relative deficits in aspects of attention, including lower than expected encoding of new information into memory (Figure 3), likely reflecting mild long-term sequelae of his protracted encephalitis. The patient participated in a feedback session in which he and his wife integrated the cognitive information with daily goals. Numerous strategies to improve memory encoding were recommended. The patient felt confident in his ability to independently integrate these recommendations in his workplace, was functioning well at home, and opted not to pursue cognitive rehabilitation therapy.

Over the ensuing year, the patient was treated with imatinib and systemic melanoma was well controlled. Unfortunately, approximately 1 year after SRS, the left temporal lesion showed increased size and contrast enhancement as well as intratumoral hemorrhage and increased surrounding edema in the left temporal lobe (Figure 2B). It was unclear whether these changes reflected radiation necrosis or recurrent melanoma. In the multidisciplinary brain metastasis tumor board, neurosurgery, radiation oncology, hematology-oncology, and neuropsychology specialists agreed that surgical resection was indicated if it could be accomplished with minimal cognitive morbidity. Neuropsychological re-evaluation was conducted and showed significant declines in memory and aspects of language compared with the assessment that had been conducted 1 year earlier (Figure 3). These findings suggested that the increased size of the lesion and surrounding edema was indeed affecting NCF.

In consultation with the patient, the decision was made to resect the lesion. A functional magnetic resonance imaging study was conducted to identify foci of critical language activity in the left hemisphere and identified a site of putative receptive language function ~1 cm from the lesion boundary. Diffusion tensor imaging was conducted during the same MRI session to identify critical fiber tracts and demonstrated the location of the arcuate fasciculus passing within 7 mm of the mass. These imaging studies were fused with the structural imaging in the surgical navigation software. Intraoperative mapping of language function demonstrated an area 1 cm superior to the lesion in which stimulation produced deficits in language comprehension. The cortex overlying the lesion was tested with stimulation mapping, and no changes were elicited in comprehension, naming, or reading. A gross total resection was achieved with pathology demonstrating recurrent melanoma. The patient remained awake throughout the procedure and demonstrated no gross deficits postoperatively.

The patient was seen for a repeat NP evaluation ~1 month after surgery. At that point, he reported good recovery of function and had returned to his part time professional role as a technical advisor to a biotechnology firm. He and his wife reported improved memory compared with the preoperative time point but acknowledged increased fatigue and reduced cognitive endurance since surgery. The evaluation demonstrated a significant improvement in memory as compared with the preoperative assessment, returning to the level of performance seen 14 months prior (Figure 3). There were also improvements in confrontation naming and verbal fluency, though there was a decline in phrase repetition. The patient participated in a short course of speech/language and cognitive rehabilitation therapy. During therapy, he developed additional strategies to support memory and assist with word-finding difficulties. At the time of this submission, he continues to function effective in his job and is fully independent in ADLs. At the most recent follow-up visit, he reported good overall QOL and denied difficulties in subjective cognitive function. This case demonstrates the potential for positive outcomes from a multidisciplinary process that integrates cognitive outcomes in the management of brain metastasis.
